# Hard X-ray ptychography for optics characterization using a partially coherent synchrotron source

**DOI:** 10.1107/S1600577520012151

**Published:** 2020-10-16

**Authors:** Thomas E. J. Moxham, Aaron Parsons, Tunhe Zhou, Lucia Alianelli, Hongchang Wang, David Laundy, Vishal Dhamgaye, Oliver J. L. Fox, Kawal Sawhney, Alexander M. Korsunsky

**Affiliations:** a Diamond Light Source, Harwell Science and Innovation Campus, Didcot OX11 0DE, United Kingdom; b Department of Engineering Science, University of Oxford, Parks Road, Oxford OX1 3PJ, United Kingdom; c Synchrotron Utilisation Section, Raja Ramanna Centre for Advanced Technology, Indore, India

**Keywords:** ptychography, synchrotron, X-ray optics, wavefront, zone-plate, compound refractive lenses

## Abstract

Ptychography is a scanning coherent diffraction imaging technique which provides high resolution imaging and complete spatial information of the complex electric field probe and sample transmission function. Its ability to accurately determine the illumination probe has led to its use at modern synchrotrons and free-electron lasers as a wavefront-sensing technique for optics alignment, monitoring and correction. Recent developments in the ptychography reconstruction process now incorporate a modal decomposition of the illuminating probe and relax the restriction of using sources with high spatial coherence. In this article a practical implementation of hard X-ray ptychography from a partially coherent X-ray source with a large number of modes is demonstrated experimentally. A strongly diffracting Siemens star test sample is imaged using the focused beam produced by either a Fresnel zone plate or beryllium compound refractive lens. The recovered probe from each optic is back propagated in order to plot the beam caustic and determine the precise focal size and position. The power distribution of the reconstructed probe modes also allows the quantification of the beams coherence and is compared with the values predicted by a Gaussian–Schell model and the optics exit intensity.

## Abstract 2

Ptychography has been developed and routinely employed as an at-wavelength metrology method on a low coherence dipole magnet beamline.

## 1. Introduction   

The short wavelength, high penetration and chemical sensitivity of hard X-rays makes them an ideal nano-probe for studying the chemical, elemental and structure of matter and has led to the development of highly brilliant sources at third-generation synchrotrons and X-ray free-electron laser facilities (XFELs). To exploit these sources the development of optics with high numerical aperture and minimal wavefront distortion is required. At shorter wavelengths the focal quality is highly sensitive to optical imperfections arising from fabrication methods, which puts stringent requirements on manufacturing tolerances and the methods to quantify them. Online or at-wavelength metrology methods are capable of measuring the wavefront error introduced by imperfect optics at the designed wavelength and are performed on the beamline or *in situ*. They are capable of overcoming optical performance factors such as beam alignment, thermal drifts and mechanical vibrations and have proven highly successful at characterizing optics (Sawhney *et al.*, 2013 ▸) as well as playing a critical role in alignment optimization (Zhou *et al.*, 2018 ▸) and aberration compensation (Laundy *et al.*, 2019 ▸).

Advancements in source coherence and emittance has led to the development of coherent diffraction imaging (CDI) techniques, capable of resolutions beyond the diffraction limit of current optics (Thibault *et al.*, 2014 ▸). Ptychography is one such technique that is able to recover the complex illumination and transmission functions of an extended object by measuring diffraction patterns at overlapping sample positions and using redundancy in the data to solve the phase problem (Thibault *et al.*, 2008 ▸). Ptychography is a beneficial characterization technique as it recovers the optics complex electric field and offers high spatial resolution compared with other methods. For example, knife-edge scanning of the intensity at the nano-scale typically overestimates the true focus size because the resolution is limited by the scanning stage and knife-edge quality (Kewish *et al.*, 2010 ▸). The reconstructed complex probe function can be propagated along the longitudinal optical axis using the Fresnel–Kirchhoff diffraction equation (Born & Wolf, 1999 ▸), in order to reveal the complete beam caustic including the focus profile and wavefront error being introduced by the optic.

Ptychography for wavefront characterization has been readily demonstrated on highly coherent insertion device beamlines at synchrotrons (Schropp *et al.*, 2011 ▸; Vila-Comamala *et al.*, 2011 ▸; Kewish *et al.*, 2010 ▸; Huang *et al.*, 2012 ▸). The small source size and large flux on these beamlines means the beam can easily be made coherent by selecting the necessary portion of the beam without detriment to the overall flux. The development of ptychography reconstruction algorithms which incorporate probe mode decomposition (Thibault & Menzel, 2013 ▸) and broad-bandwidth radiation (Enders *et al.*, 2014 ▸) relaxes the condition of high transverse and longitudinal coherence respectively. Previous studies into ptychography with partial coherence have generally been conducted using numerical simulations (Burdet *et al.*, 2015 ▸) and for insertion devices where a large portion of the beam is already highly coherent (Stachnik *et al.*, 2015 ▸; Rose *et al.*, 2018 ▸), but limited success has so far been demonstrated with truly partially coherent sources (Macrander *et al.*, 2017 ▸).

In this article we report on the successful implementation of ptychography using the B16 Test beamline at Diamond Light Source, a partially coherent source with a large source size and relatively low flux. Ptychography measurements are performed using a Siemens star test sample to recover the complex illumination and object functions from focused beams produced by either a Fresnel zone plate (FZP) or a beryllium compound refractive lens (CRL). The recovered probe is back propagated to generate a beam caustic and reveal the precise focal plane position and focus size. In addition to this the power distribution from the reconstructed probe modes is used to quantify the normalized degree of coherence and is compared with the values predicted by a Gaussian–Schell model using the optics exit intensity.

## 2. Partially coherent ptychography   

Ptychography is an imaging technique that uses diffraction patterns from overlapping sample positions to solve the phase problem and give a complete description of the sample and probe illuminating it. A sample is illuminated with a probe and scanned transversely, in order to produce a set of diffraction patterns *I*_*i*_ at each position 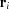 given by (Thibault *et al.*, 2008 ▸) 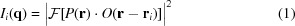
where 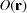 is the samples complex transmission function, 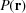 is the probes complex illumination function and 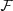 denotes the Fourier transform with the corresponding reciprocal space coordinate 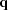. Successful reconstruction depends on two oversampling criteria being satisfied. The first is that the scanning step size must be greater than 33% of the beam size between successive diffraction patterns (Bunk *et al.*, 2008 ▸), and the second is that the illuminating beam size must satisfy the condition (Edo *et al.*, 2013 ▸) 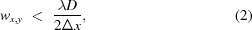
where λ is the X-ray wavelength, *D* is the sample to detector distance and Δ*x* is the detector pixel size. Since we know our source to be partially coherent, we will consider a multi-modal ptychography approach (Thibault & Menzel, 2013 ▸) and decompose the probe function 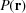 as a sum of independent spatially coherent states *E*_*n*_. If we assume the emission from each electron in the synchrotron storage ring is independent of one another, the transverse coherence is a measure of the correlation between two points on the resulting electric field at two spatially separated positions. The cross-spectral density function for a partially coherent, stationary quasi-monochromatic electric field was first derived by Mandel & Wolf (1995 ▸) and applied explicitly to synchrotron and free-electron laser studies by Vartanyants & Singer (2010 ▸); it can be written as a sum of independent spatially coherent states given by
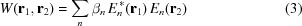
where 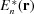 is the complex conjugate of the electric field, β_*n*_ are eigenvalues and 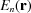 are eigenfunctions, which form an orthogonal set that satisfies the Fredholm integral equation of the second kind given by
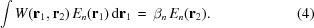
The eigenvalues β_*n*_ are positive and real; from equation (3) they represent and are sometimes referred to as modal weights, equal to the power contribution of each individual mode. From Vartanyants & Singer (2010 ▸) it was also shown that the synchrotron source can be accurately represented by a Gaussian–Schell model, which allowed solutions to equation (4) with the eigenfunctions 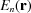 described by Gaussian–Hermite modes (not shown) and the eigenvalues β_*n*_ following a power-law dependence given by 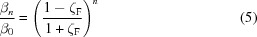
where ζ_F_ is the total normalized degree of transverse coherence (fitted) and is equal to the percentage of coherent flux; it lies in the range 0 ≤ ζ ≤ 1 with complete spatial coherence when ζ → 1 and complete spatial incoherence when ζ → 0. A direct calculation of the normalized degree of coherence from the eigenvalues was also derived by Khubbutdinov *et al.* (2019 ▸) and is given by 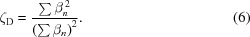
However, as will be shown later in Section 7, the fitted normalized degree of coherence of equation (5) is much more accurate at determining values for experimental data than the direct normalized degree of coherence of equation (6).

The ptychography phase retrieval algorithm then iterates the diffraction dataset of equation (1) between the real and reciprocal space and applies a probe and modulus constraint, respectively, in each. For the proceeding reconstructions we will use the Difference Map and Maximum Likelihood phase retrieval algorithms; detailed descriptions of their use is beyond the scope of this paper but further information can be found elsewhere (Thibault *et al.*, 2009 ▸; Thibault & Guizar-Sicairos, 2012 ▸).

## 3. Source coherence theory   

In order to validate coherence results recovered from ptychography we require an estimation of the normalized degree of coherence at the optics entrance plane. The beam’s transverse coherence depends on the source size and divergence, values of which for both can be calculated from the complex electric field recovered during ptychography. The aperture formed by the slits 18.4 m downstream of the bending magnet defines a secondary source and requires accurate determination. For both focusing optics used, the focal size is limited primarily by the demagnified source size, as the diffraction-limited contribution is negligible, meaning the slit size is given by
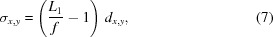
where σ_*x*,*y*_ is the root mean square slit size, *L*_1_ is the slits-to-optic distance, *f* is the optics focal length and *d*_*x*,*y*_ is the size of the image formed. To determine beam divergence from the secondary source the electric field recovered from ptychography is back propagated along the optical axis to the optic plane. From the slit size determined using equation (7) and the beam size at the optic plane, the secondary source divergence can be calculated by (Cai *et al.*, 1997 ▸)
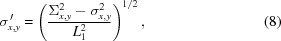
where Σ_*x*,*y*_ is the measured beam size at the optic plane and all other values are as stated previously. With values for the source size and divergence, the secondary source can be modelled as in the previous section by a Gaussian–Schell model (Vartanyants & Singer, 2010 ▸). This assumes the slits act as a plane two-dimensional source that is spatially uniform and gives the coherence length at the slits by 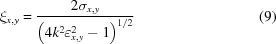
where *k* is the wavenumber and ∊_*x*, *y*_ = 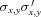 is the source emittance. Given values for the coherence length and source emittance, the degree of coherence *q*_*x*,*y*_ and hence normalized degree of coherence ζ_*x*, *y*_ are also given by (Vartanyants & Singer, 2010 ▸) 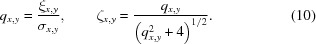
These values should remain constant along the beam path up to the optic plane, and the transverse product of the normalized degree of coherence ζ = ζ_*x*_ζ_*y*_ will serve as the comparative value found with the ptychography reconstruction.

## 4. Experimental method   

**Figure 1:**
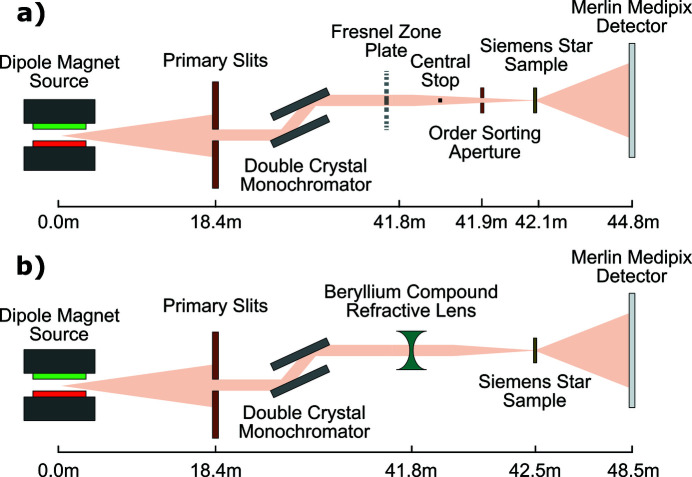
Schematic of the experimental setup and distances for (*a*) the Fresnel zone plate and (*b*) the beryllium compound refractive lens. Distances are not to scale and diffraction patterns are collected in the far-field using a Merlin Medipix detector.

The measurements were performed on the B16 Test beamline at Diamond Light Source, a bending dipole magnet beamline that provided an unfocused, monochromatic beam (Sawhney *et al.*, 2010 ▸). To improve the source coherence, primary slits 18.4 m from the source were nominally set at 50 µm (h) × 50 µm (v), and a Si(111) double-crystal monochromator was used to select an energy of 10 keV and 15 keV for the FZP and CRL setups, respectively. A schematic of both experimental setups is shown in Fig. 1 ▸. In both setups a 3 µm-thick gold Siemens star on a 0.3 µm-thick silicon nitride substrate was used as the strongly diffracting test sample. The Siemens star had a diameter of 200 µm and inner spoke separation of 1 µm as the smallest feature size. The sample was mounted on linear Attocube piezo stages with 10 nm precision and scanned transversely to the beam; this was done in a spiral fashion ensuring at least 33% overlap in an effort to eliminate reconstruction artefacts and satisfy the first oversampling condition. The oversampling condition set by the illumination probe size and described by equation (2) was satisfied by performing ptychography scans along the beam path until satisfactory reconstructions were obtained and the reconstructed beam size was smaller than that required. For the experimental setups shown in Fig. 1 ▸ the smallest allowed probe sizes were 3.1 µm and 4.5 µm for the FZP and CRL setups, respectively.

## 5. Fresnel zone plate   

The first optic characterized was a tungsten FZP on a silicon nitride substrate which featured a 245 µm diameter, 123 nm outer ring width and a 60 µm-diameter fixed central stop. An order sorting aperture (OSA) with a 60 µm diameter was mounted 184 mm downstream of the optic. At 10 keV this gave a first-order focal length of 243 mm; the Siemens star test object was positioned slightly downstream of the focus. A high-dynamic-range second-generation Merlin Medipix detector with a 256 × 256 pixel field of view, and 55 × 55 µm pixel size was mounted in the far-field approximately 2.72 m downstream of the sample. At the optimized probe position a ptychography scan was performed over an area of 20 µm × 20 µm with a step size of 0.6 µm and individual exposure time of 1 s, which generated 1109 diffraction patterns for ptychographic reconstruction.

The diffraction data were cropped to a region of interest around the optical axis measuring 180 × 180 pixels which included only statistically relevant counts. Dead detector pixels were also disregarded from the ptychographic reconstruction by generating a detector mask based on the integrated diffraction data stack. Ptychographic reconstructions were performed using the *ptypy* framework (Enders & Thibault, 2016 ▸) so that we could utilize reconstruction parallelization with a wide range of algorithms and additional options. In order to achieve a convergent reconstruction incoherent modes were required (Thibault & Menzel, 2013 ▸); these were included until the power in the final mode tended to zero (as in Fig. 8), which occurred with the use of 30 modes. An initial reconstruction with 1000 iterations of the Difference Map algorithm (Thibault *et al.*, 2009 ▸) was used, followed by a refinement procedure using 1000 iterations of the Maximum Likelihood algorithm (Thibault & Guizar-Sicairos, 2012 ▸).

**Figure 2:**
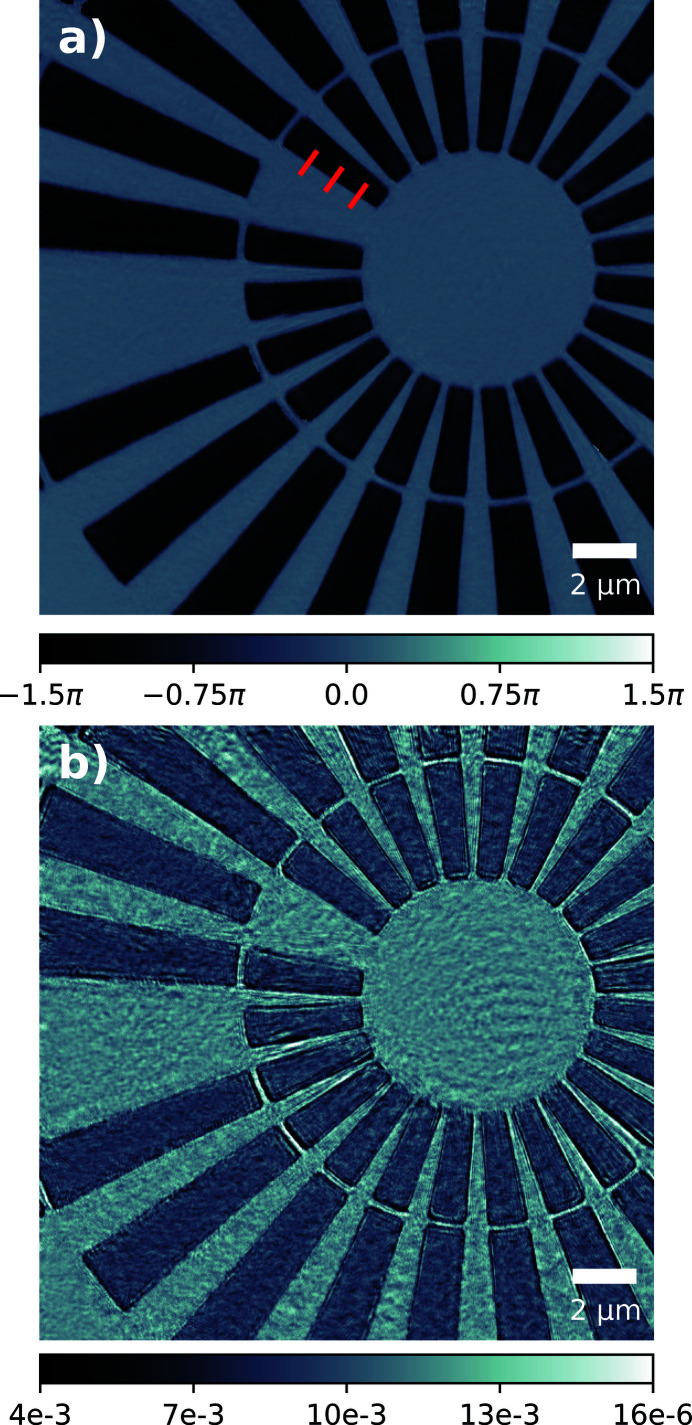
Ptychographic reconstruction of the Siemens star object (*a*) phase and (*b*) amplitude using a Fresnel zone plate illumination at 10 keV; units of phase are in radians (rad) and amplitude are in arbitrary units (a.u.). Red markings denote where line profiles were taken along sharp features in order to quantify the obtained resolution; plots of these can be found in the supporting information.

**Figure 3:**
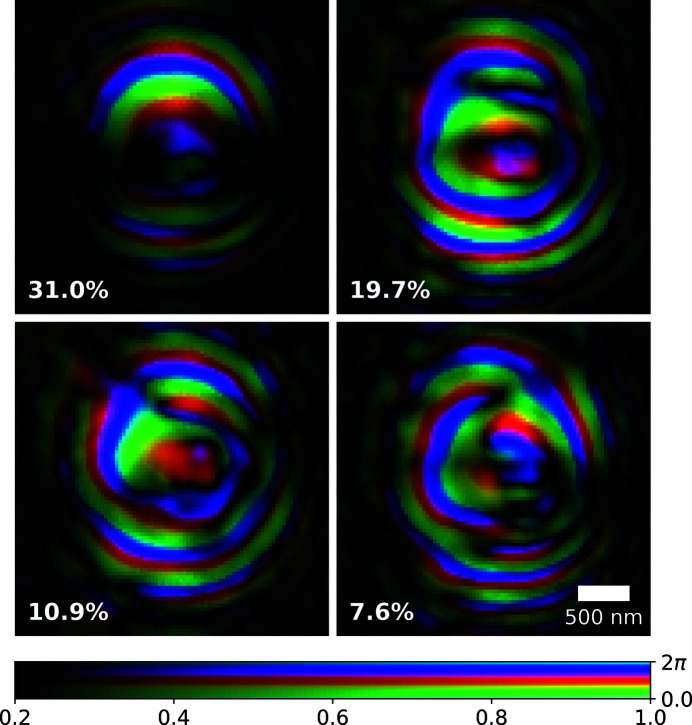
Complex representation of the first four dominant probe modes (after orthogonalization) with a Fresnel zone plate illumination; the relative mode power is also given in the bottom left corner. Brightness and hue represent amplitude and phase, respectively.

For the experimental geometry outlined, an effective pixel size of 35 nm was achieved; the reconstructed Siemens star phase and amplitude are shown in Figs. 2(*a*) and 2(*b*) ▸, respectively. The measured phase offset introduced by the Siemens star is 1.31π which is within fabrication tolerances of the designed value of 1.44π at 10 keV. The reconstructed illumination probe had the same pixel size and was orthogonalized according to the Gram–Schmidt process (Li *et al.*, 2016 ▸). The first four dominant modes are shown in Fig. 3 ▸ after orthogonalization; their relative power to one another is small and there is no particular mode that concentrates most of the power unlike highly coherent sources. The relative power for all modes is also plotted in Fig. 8.

**Figure 4:**
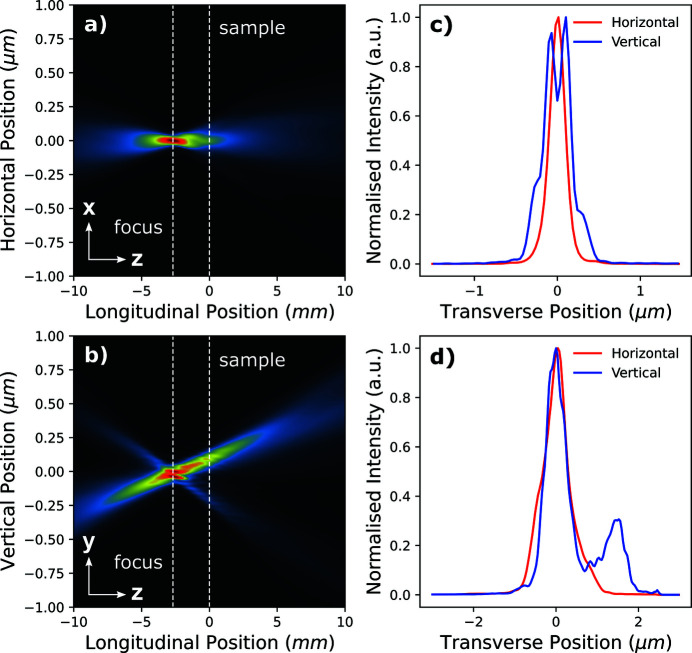
Propagation of the reconstructed probe from Fig. 3 ▸, with weighted intensity contributions from each probe mode. (*a*) The beam intensity integrated along the *x*-plane, showing the vertical beam waist in the *yz*-plane. (*b*) The beam intensity integrated along the *y*-plane, showing the horizontal beam waist in the *yx*-plane. The sample position and focal plane are labelled by the white dashed line at 0.0 mm and −2.7 mm, respectively. (*c*) Horizontal and vertical profiles of the intensity of the beam at the focus position. (*d*) Horizontal and vertical profiles of the intensity of the beam at the sample position.

Since the ptychographic method recovers a complex wavefield for the probe at the position of the Siemens star, we are able to use the Fresnel–Kirchhoff diffraction formula to numerically propagate along the optical axis and generate a beam caustic. In accordance with equation (3), all complex probe modes were propagated individually ±10 mm along the longitudinal axis in 20 µm steps and the contributions to the intensity at each plane were scaled and summed accordingly. The focal plane was defined as the plane which maximized peak intensity, and in this case was determined to be 2.7 mm upstream of the object horizontally and vertically. The horizontal and vertical beam caustic intensity profiles (integrated along the vertical and horizontal, respectively) are shown, and also label the sample and focal planes in Fig. 4 ▸. Horizontal and vertical line profiles taken at the focal plane shown in Fig. 4(*c*) ▸ reveal the root mean squared (RMS) focal size to be 93.8 nm (h) × 173.7 nm (v). The expected focus size should be limited by the source demagnification according to equation (7) and suggests the RMS size of the secondary source formed by the primary slits is 8.9 µm (h) × 16.6 µm (v).

## 6. Beryllium compound refractive lens   

The second optic characterized was a beryllium CRL with 98 lenses, a geometric aperture of 1 mm and a radius of curvature of 200 µm. The experimental setup was similar to the FZP except no central stop or OSA were used and an energy of 15 keV was selected which gave the CRL a focal length of 690 mm. In addition, a third-generation Merlin Medipix detector with the same 55 µm × 55 µm pixel size but larger 512 × 512 pixel field of view was employed and mounted slightly further downstream 6 m from the sample. At the optimized probe position, a ptychography scan was performed over an area of 12 µm × 12 µm with a step size of 0.3 µm and individual exposure time of 0.2 s, which generated 2581 diffraction patterns for ptychographic reconstruction.

**Figure 5:**
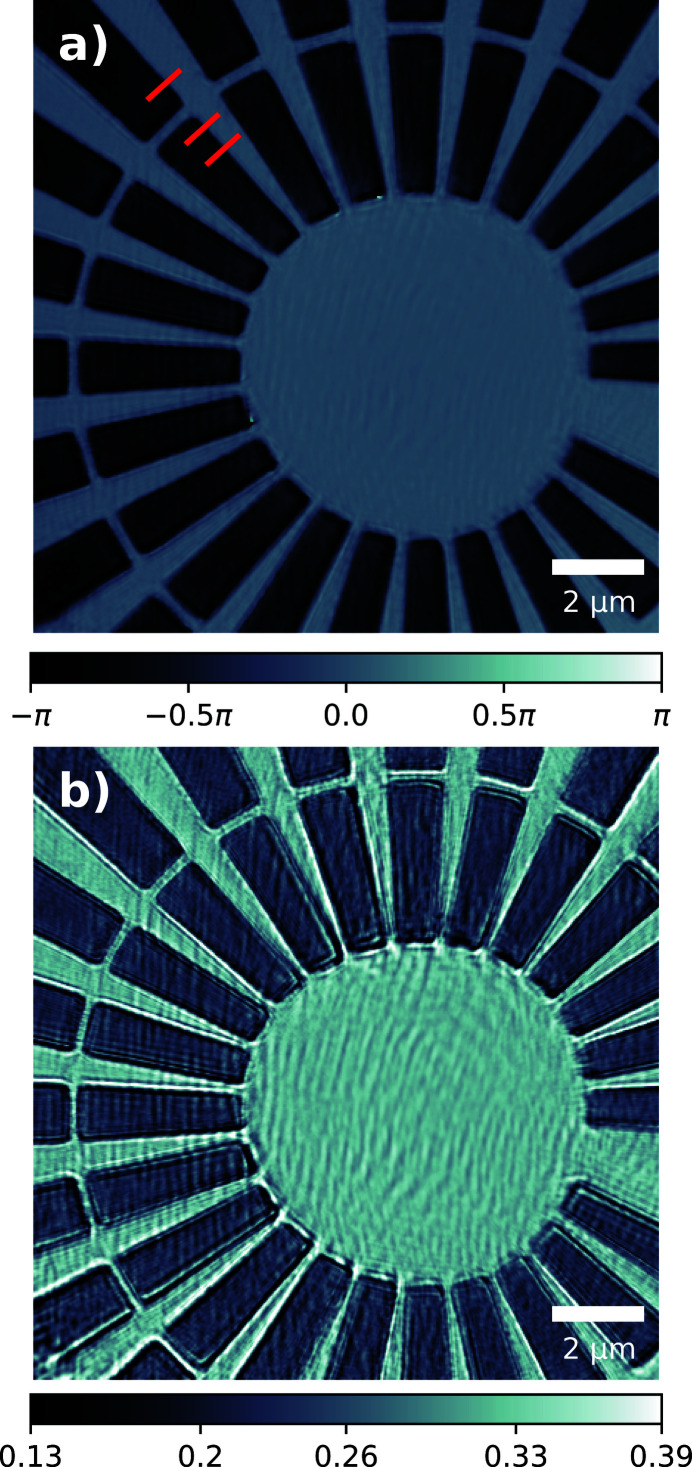
Ptychographic reconstruction of the Siemens star object (*a*) phase and (*b*) amplitude using the beryillium compound refractive lens illumination at 15 keV; units of phase are in radians (rad) and amplitude are arbitrary units (a.u.). Red markings denote where line profiles were taken along sharp features in order to quantify the obtained resolution; plots of these can be found in the supporting information.

**Figure 6:**
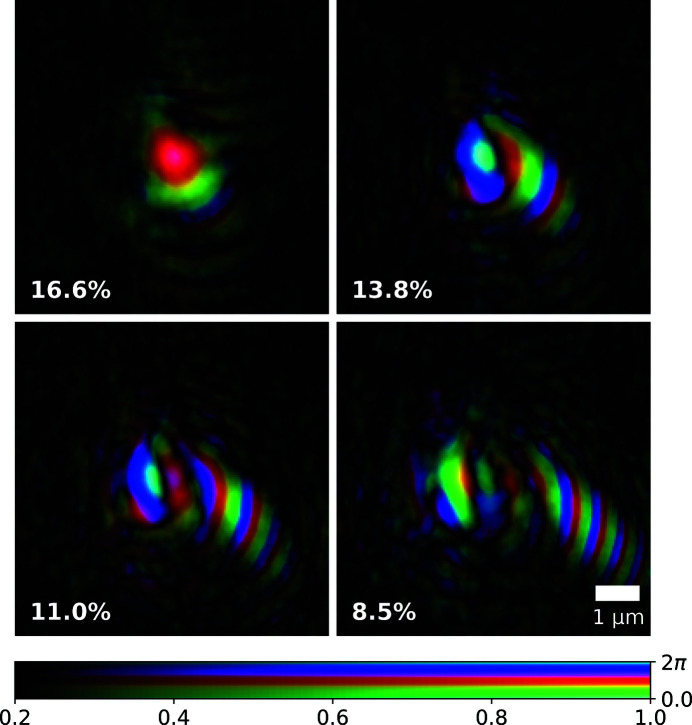
Complex representation of the first four dominant probe modes (after orthogonalization) with beryillium compound refractive lens illumination. The relative mode power is also given in the bottom left corner; brightness and hue represent amplitude and phase respectively.

As before, the diffraction data were cropped to a region of interest around the optical axis measuring 280 × 280 pixels which included only statistically relevant counts. The rest of the data preparation and ptychographic reconstruction followed the exact same process as outlined in the previous section on the FZP, and resulted in an effective pixel size of 32 nm. The reconstructed Siemens star phase and amplitude are shown in Figs. 5(*a*) and 5(*b*) ▸, respectively. The phase offset introduced by the Siemens star is 0.89π which is within fabrication tolerances of the designed value of 0.96π at 15 keV. The first four dominant modes are shown in Fig. 6 ▸ after orthogonalization; their relative power to one another is small and there is no particular mode that concentrates most of the power unlike highly coherent sources. The relative power for all modes is also plotted in Fig. 8.

**Figure 7:**
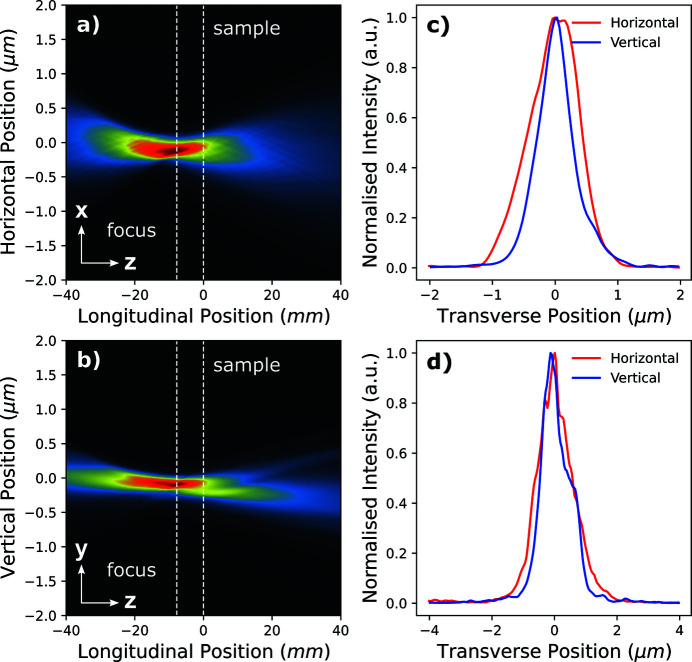
Propagation of the reconstructed probe from Fig. 6 ▸, with weighted intensity contributions from each probe mode. (*a*) The beam intensity integrated along the *x*-plane, showing the vertical beam waist in the *yz*-plane. (*b*) The beam intensity integrated along the *y*-plane, showing the horizontal beam waist in the *yx*-plane. The sample position and focal plane are labelled by the white dashed line at 0.0 mm and −9.1 mm, respectively. (*c*) Horizontal and vertical profiles of the intensity of the beam at the focus position. (*d*) Horizontal and vertical profiles of the intensity of the beam at the sample position.

The complex probe modes were numerically propagated using the Fresnel–Kirchhoff diffraction formula along the optical axis in order to generate a beam caustic. In accordance with equation (3), all complex probe modes were propagated individually ±40 mm along the longitudinal axis in 80 µm steps and the contributions to the intensity at each point were summed. The focal plane was determined to be 9.1 mm upstream of the object in both the horizontal and vertical plane. The horizontal and vertical beam caustic intensity profiles (integrated along the vertical and horizontal, respectively) are shown in Fig. 7 ▸ and label the sample and focal planes. Horizontal and vertical line profiles taken at the focal plane, shown in Fig. 7 ▸(*c*), reveal the RMS focal size to be 387.7 nm (h) × 300.2 nm (v). The expected focus size should be limited by the source demagnification according to equation (7) and suggests the RMS size of the secondary source formed by the primary slits is 12.8 µm (h) × 9.9 µm (v).

## 7. Results   

Often the quality and reliability of the reconstructed illumination probe is evaluated using the reconstructed object, and in Figs. 2 ▸ and 5 ▸ there is good agreement with known feature sizes from the fabrication design and scanning electron microscope images (not shown). The spatial resolution of the reconstructions is sufficiently high to visually distinguish the 1 µm feature separating the innermost radial spokes. To quantify the resolution further, the sharp edge of one of the spokes was chosen, along which error-like line profiles were taken. The position of these is marked by red lines in Figs. 2 ▸(*a*) and 5 ▸(*a*), with the corresponding line profiles included in the supporting information. The profiles were extrapolated and differentiated such that a Gaussian profile could be fitted; this revealed an average full width at half-maximum size (and, hence, resolution) of 81.1 nm and 80.7 nm for the FZP and CRL setups, respectively. The reconstructed pixel sizes for both optic setups improves the imaging capability of the beamline, which typically only offers resolutions of 300 nm due to the relatively large source size and availability of optics.

To quantitatively assess the coherent properties of the setups we compare values of the total degree of transverse coherence recovered from the modal decomposition of the probe modes and the values calculated based on the experimental parameters. Firstly, the probe recovered for each optic is back propagated to the optics plane and line profiles are taken of the intensity; figures are included in the supporting information. By considering the optics to be thin elements we can calculate the beam divergence from the primary slits according to equation (8) and using the previously recovered values for the primary slits size. Due to the beam stop included in the FZP setup an accurate estimation of the beam size at the optics plane is difficult, so the divergence calculated for the CRL is used in its place. Using values for the source size and diveregence, the source coherence length and degree of coherence can be calculated according to equations (9) and (10), respectively; all values used in the calculation are displayed in Table 1 ▸ for convenience.

**Figure 8:**
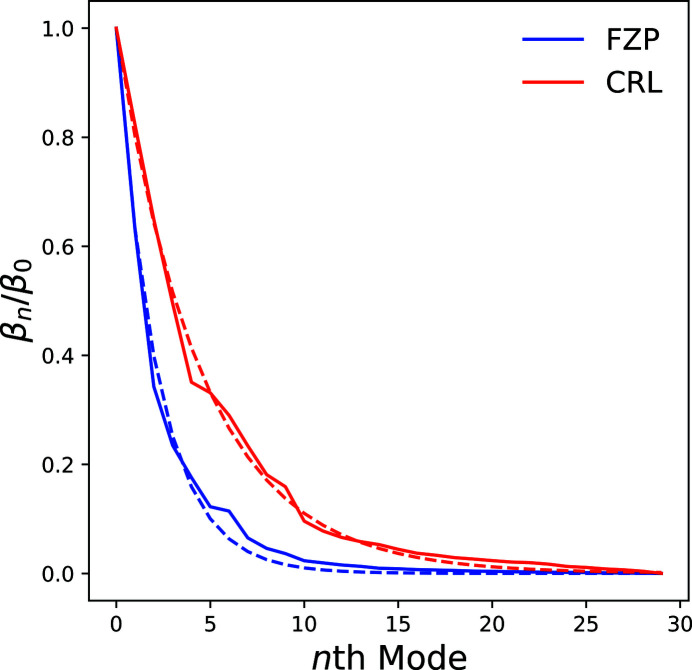
The ratios of the reconstructed probe eigenvalues β_*n*_ to the dominant eigenvalue β_0_ after orthogonalization plotted against modal number; the dashed line is a fitted function according to equation (5) in order to determine the degree of transverse coherence.

In order to compare the values recovered from the modal decomposition of the probe, the ratios of the eigenvalues β_*n*_ to the dominant eigenvalue β_0_ are plotted against the modal number, and a linear fractional function with a power-law dependence according to equation (5) is fitted, as shown in Fig. 8 ▸. This found that the normalized degree of coherence (and hence proportion of coherent flux) for the FZP setup is ζ_F_ = 0.217 and for the CRL setup ζ_F_ = 0.114. In addition to this, a direct computation of the normalized degree of transverse coherence according to equation (6) was computed and found values for the FZP setup to be ζ_D_ = 0.162 and for the CRL setup ζ_D_ = 0.083. As can be seen in Table 1 ▸, the fitted values are much closer to the predicted values primarily because the experimentally obtained eigenvalues do not follow an exact power-law dependence. As expected, the CRL setup produces a relatively lower degree of coherence due to the higher energy used. In both cases the proportion of coherent flux is low and a large number of modes are required to model the source properly, demonstrating the partial coherent nature of the X-ray illumination.

## 8. Conclusion   

We have demonstrated a practical implementation of ptychography using a partially coherent X-ray source with a large number of modes in the reconstruction. Ptychography scans were performed using a FZP at 10 keV and a CRL at 15 keV as the illumination probes. The electric field recovered from each of these optics could be back propagated using the Fresnel–Kirchhoff diffraction equation in order to determine the complete beam caustic and measure the precise focal size and position. The normalized degree of coherence was found for both optics using the intensity at the optic plane as well as directly from the decomposed probe mode eigenvalues. Both of theses values showed close agreement and allowed for accurate quantification of the proportion of coherent flux incident on the optics. The reconstructed complex sample transmission function resolved sample features below that normally offered by the beamline and also provides both phase and absorption information. Unlike other wavefront metrology techniques, ptychography is able to correct for relatively common issues in post-processing such as sample positioning errors and optical vibrations. In future developments of the technique on B16, we plan to optimize the primary aperture settings in order to fully illuminate the geometric aperture of optics and recover the complete two dimensional wavefront error. We have shown that ptychography is no longer a specialist technique restricted to highly coherent synchrotron sources, but is now being routinely employed and developed as a complementary metrology technique on the B16 Test beamline at Diamond Light Source. Partial coherent ptychography is now a realized technique that extends the imaging capabilities of existing beamlines without the need for expensive optics and major equipment upgrades.

## Supplementary Material

Supporting information file. DOI: 10.1107/S1600577520012151/gb5108sup1.pdf

## Figures and Tables

**Table 1:** Beam coherence determined for the Fresnel zone plate at 10 keV and the beryllium compound refractive lens at 15 keV using the beam divergence calculated from the slit size and the optics exit intensity at a position 23.4 m from the slits

	FZP at 10 keV		CRL at 15 keV	
	*x*	*y*	*x*	*y*
Secondary source or slits size, σ_*x*, *y*_	8.91 µm	16.5 µm	12.8 µm	9.91 µm
Beam divergence from slits, 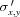	1.54 µrad	0.699 µrad	1.54 µrad	0.699 µrad
Coherence length at slits, ξ_*x*, *y*_	18.4 µm	54.5 µm	9.06 µm	60.6 µm
Degree of coherence at slits, *q*_*x*, *y*_	0.879	1.40	0.301	2.60
Effective coherence length at optic, Ξ_*x*, *y*_	59.5 µm	64.4 µm	25.9 µm	79.2 µm
Designed optics aperture, *A*_*x*, *y*_	245 µm	245 µm	474 µm	474 µm
Normalized degree of coherence, ζ_*x*, *y*_	0.402	0.574	0.149	0.793
Total normalized degree of coherence, ζ	0.231		0.118	
