# Supplementary material for: Hard X-ray ptychography for optics characterization using a partially coherent synchrotron source
Source: J Synchrotron Radiat. 2020 Oct 16;27(Pt 6):1688–95. doi: 10.1107/S1600577520012151 (PMC7642961; doi:10.1107/S1600577520012151)
Supplement: Supplementary file 1 [file s-27-01688-sup1.pdf]

Supporting Information: Hard X-ray Ptychography for Optics Characterization Using a Partially Coherent Synchrotron Source

T.E.J. Moxham[1,2,*], A. Parsons[1], T. Zhou[1], L. Alianelli[1], H. Wang[1], D. Laundy[1], V. Dhamgaye[1,3], O.J.L. Fox[1], K. Sawhney[1] and A.M. Korsunsky[2]

[1]*Diamond Light Source, Harwell Science Innovation Campus, Didcot, OX11 0DE, U.K*
[2]*Department of Engineering Science, University of Oxford, Parks Road, Oxford, OX1 3PJ, U.K*
[3]*Synchrotron Utilisation Section, Raja Ramanna Centre for Advanced Technology, Indore, India*

S1: Optic's Exit Intensity

In order to characterise the coherent properties of the beam it is necessary to evaluate the intensity profile of the incident beam on the optic. From the beam size at the optic plane, the secondary source (or slit) size and the distance between the two planes, the divergence and hence coherence length can be calculated. The probe recovered during ptychography reconstruction was first back propagated to the focal plane, and then further back propagated a distance equal to the focal length of each optic. Line profiles were taken through the horizontal and vertical planes at approximately the center, to which Gaussian profiles were fitted in order to extract the RMS value for each transverse direction. The RMS beam size at the CRL optic plane was found to be $38.2\mu m\ (\mathrm{h}) \times 19.1\mu m\ (\mathrm{v})$. Due to the beam stop present in the FZP setup, an accurate estimation of the beam size at the optic plane was unobtainable, the divergence calculated from the CRL setup was used instead for coherence calculations. All results for intensity at the optic plane and transverse profiles are shown in Fig. S1.

S2: Ptychography Resolution Analysis

An estimate of the actual resolution rather than the ptychography reconstructed pixel size is made by taking line profiles across sharp features of the sample. The image was first interpolated by a factor of 4 in order to include a statistically significant number of points along the curve. Then error like line profiles were taken across the sharp edges of the Siemens star spoke, as marked by red lines in Fig. 2a and 5a in the main paper. These were differentiated in order to generate a peak profile to which a Gaussian was fitted in order to estimate the full width half maximum (FWHM) which we take to be the estimated spatial resolution. Three line profiles were taken on the sample and the average FWHM spatial resolution was found to be $81.1nm$ and $80.7nm$ for the FZP and CRL respectively. All results for the line profiles across the sharp edges and the subsequent differentiation and Gaussian fitting are shown in Fig. S2.

* corresponding author: thomas.moxham@eng.ox.ac.uk

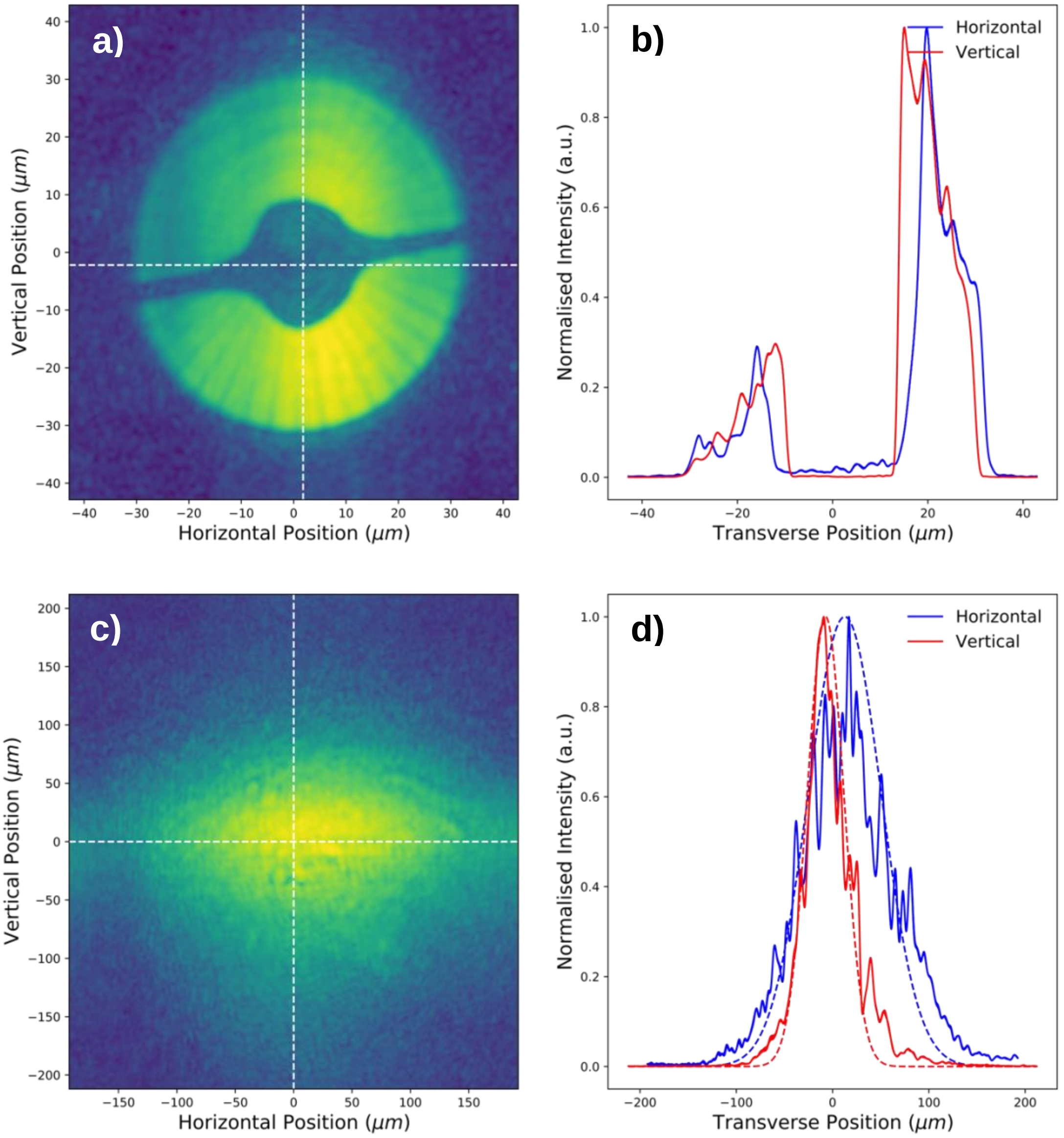

Figure S1 *(Left) 2D intensity at the optics plane plotted on logarithmic scale for a) the Fresnel zone plate and c) the Beryllium compound refractive lens. (Right) 1D normalised intensity slice taken along the white dashed line shown in the 2D plots for b) the Fresnel zone plate and d) the beryllium compound refractive lens. Dotted lines are the fitted Gaussian profile*

* corresponding author: thomas.moxham@eng.ox.ac.uk

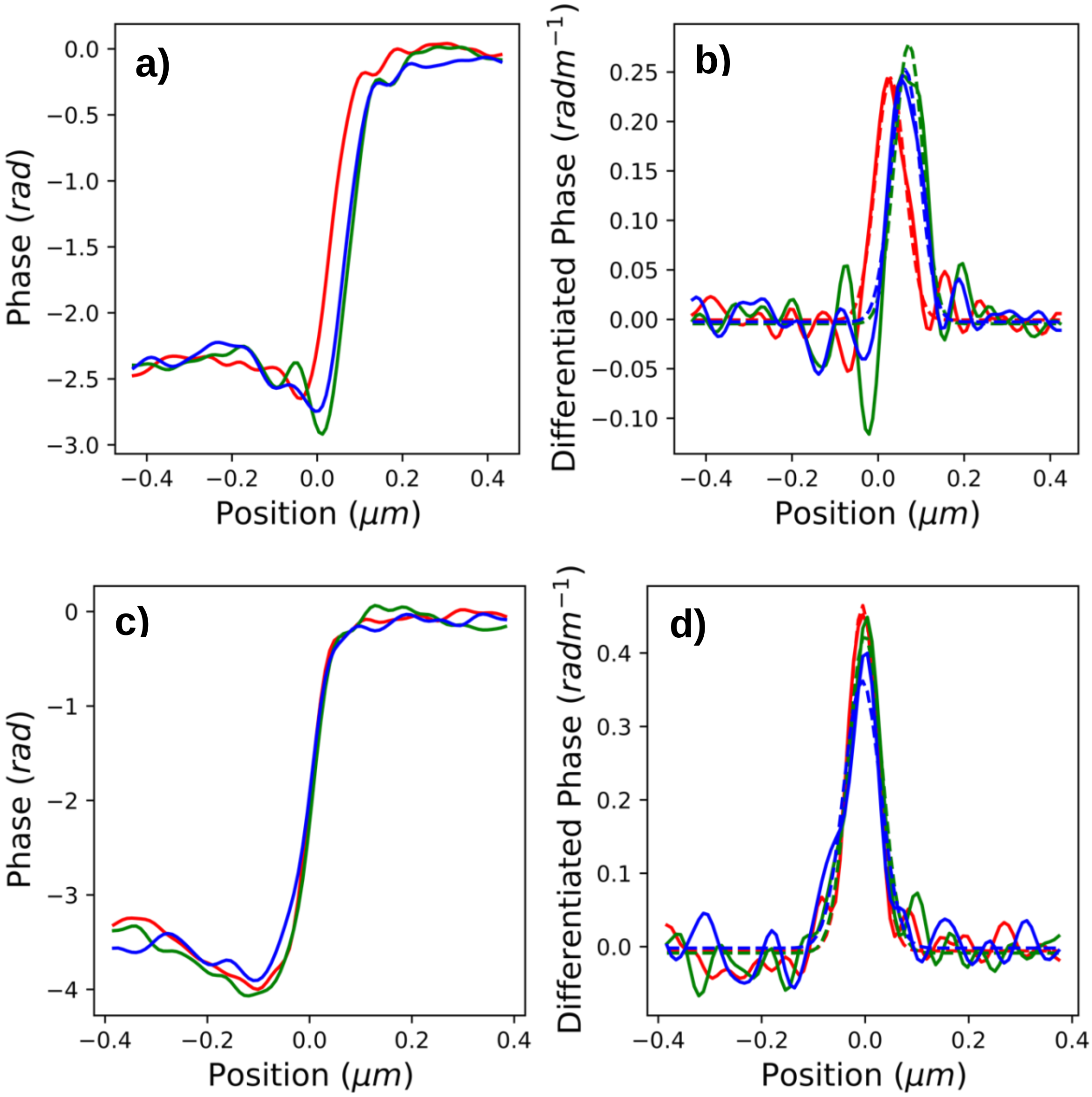

Figure S2 *(Left) Error like line profiles taken across sharp edges of the ptychography reconstructed phase for a) the Fresnel zone plate and c) the Beryllium compound refractive lens. (Right) The corresponding differentiated line profiles with Gaussian shown by the dashed line for b) the for b) the Fresnel zone plate and d) the Beryllium compound refractive lens.*

* corresponding author: thomas.moxham@eng.ox.ac.uk
